# A comprehensive evaluation of two sample treatment procedures for the determination of emerging and historical halogenated flame retardants in biota

**DOI:** 10.1007/s11356-020-10966-y

**Published:** 2020-10-07

**Authors:** Imma Tolosa, David Huertas, Sarah Choyke, Sylvia Sander, Yann Aminot

**Affiliations:** 1 IAEA Environment Laboratories, 4a Quai Antoine 1er, 98000 Monaco, Principality of Monaco; 2 Present Address: IFREMER, Laboratory of Biogeochemistry of Organic Contaminants, Rue de l’Ile d’Yeu, BP 21105, 44311 Nantes Cedex 3, France

**Keywords:** Halogenated flame retardants, Biota, Clean-up, GC-MS/MS, LC-MS/MS, Emerging contaminants

## Abstract

Two different sample preparation protocols for the determination of 37 emerging and historical halogenated flame retardants (HFRs) in marine tissues were compared with regards to extraction recovery, lipid removal efficiency, repeatability, reproducibility, and ability to measure sub-ng g^−1^ (dry weight) concentrations in marine biota. One method involved a purification step using gel permeation chromatography (GPC) followed by a HPLC fractionation step on a Partisil amino-cyano normal phase (GPC-Partisil procedure) and the other more traditional method was based on sulphuric acid treatment followed by silica column fractionation (H_2_SO_4_-silica procedure). The samples were analysed by gas chromatography (GC) and liquid chromatography (LC) tandem mass spectrometry (MS/MS). Sample fractionation in both methods enabled unique sample preparation procedures to isolate the GC from the LC amenable compounds. Both methods could remove > 99% of the lipids which was necessary prior to GC- and LC-MS/MS analyses. The majority of the target compounds (70%) had acceptable recoveries between 60–120% for both methods. However, the sulphuric acid treatment resulted in the degradation of the TBP-AE and the silica column fractionation resulted in the loss of BEH-TEBP and the elution of PBB-Acr and TBBPA-BME in the unsuitable fraction. High recoveries of DBE-DBCH (α+β), EHTBB, BTBPE, BEH-TEBP, and PBB-Acr were attributed to matrix effects, suggesting the need to use isotope-labelled surrogate standards of the target compounds. The optimisation of the silica column chromatography, GPC, and Partisil fractionation is described and discussed to afford easy implementation of the method. The method using GPC followed by Partisil fractionation is more efficient and more reproducible than the sulphuric acid-silica procedure. The application of this method to marine biota reference materials revealed the presence of relatively high concentrations of DBE-DBCH isomers and BDE-47 in fish samples. The method detection limits comply with the recommendations of the European Commission.

**Electronic supplementary material:**

The online version of this article (10.1007/s11356-020-10966-y) contains supplementary material, which is available to authorized users.

## Introduction

The marine environment and its biological resources are threatened by the presence of anthropogenic chemicals originating from industrial, domestic, and agricultural applications. Halogenated flame retardants (HFRs), including historically used polychlorinated biphenyls (PCBs), polybrominated diphenyl ethers (PBDEs), and a myriad of other emerging HFRs with similar physiochemical properties, are of particular concern due to their persistence, bioaccumulation, and toxicity (Cruz et al. 2015; Bilal et al. 2019; Pittinger and Pecquet 2018; Xiong et al. 2019). To date, at least 75 different brominated flame retardants (BFRs) have been produced, with the major used BFRs being PBDEs, hexabromocyclododecanes (HBCDDs), and tetrabromobisphenol A (TBBPA) (Birnbaum and Staskal 2004). In 2012, the cumulative global production of BFRs exceeded 200,000 tons per year and chlorinated flame retardants had even higher production volumes (Bergman et al. 2012). Emerging and historical HFRs have been found worldwide in sediment and throughout trophic levels in marine organisms (Aznar-Alemany et al. 2018). Biota samples, like fish or mussels, make good tracers for monitoring the presence of these hydrophobic anthropogenic chemicals in the water column. In fact, biota samples are among the relevant candidate matrices proposed by environmental agencies, such as the Water Frame Directive (WFD) and Marine Strategy Frame Directive (MSFD) of the European Union; the Convention for the Protection of the Marine Environment of the North-East Atlantic (OSPAR Convention); and the Baltic Marine Environment Protection Commission (Helsinki Commission, HELCOM). In this framework, the European Commission recommended monitoring for trace levels of BFRs in seafood (European Commission, 2014), and thus, emerging HFRs were included in the Norman network list of emerging contaminants (Network of reference laboratories, research centres, and related organisations for monitoring emerging environmental substances, NORMAN 2016). It is challenging to develop a multi-analyte method for all the listed compounds in the NORMAN database and yet, the EU environmental monitoring programs rely on robust and accurate methods to screen for trace levels of contaminants in biota. The difficulty inherent to these matrices comes from the need to separate the analytes, which occur at the picogram level, from the co-extracted constituents of the matrix, particularly lipids, which are found at the milligram level. Additionally, the lipids can cause signal suppression or enhancement and matrix interference during sample analysis. Current purification methods rely on phase separation of sulphuric acid (H_2_SO_4_)–treated extracts or molecular size fractionation by gel permeation chromatography (GPC), while fractionation methods rely on normal phase column chromatography. These techniques can be combined to achieve greater lipid removal from the extracts prior to analysis. However, due to the wide range of physicochemical properties of the HFRs, the transferability of methods between compounds with previously validated levels of PCBs and PBDEs is rather limited as a priori knowledge of the elution behaviour or stability to sulphuric acid treatment is not guaranteed.

The objective of this work was to develop and compare two sample preparation methods for the determination of historical and emerging HFRs in marine tissue by isolating the gas chromatography (GC) from the liquid chromatography (LC) amenable compounds. Current methods were developed to analyse historical BFRs, such as PBDEs, which are stable to H_2_SO_4_ treatment and are GC amenable (de Boer et al. 2001). As the list of emerging HFRs increases, the existing procedures need to be assessed, optimised, and modified for emerging HFRs, which may include LC amenable compounds (Papachlimitzou et al. 2012). In this framework, we aim to develop and compare two sample preparation procedures, which are able to remove the sample matrix and separate the GC from the LC amenable compounds. The advantage of separating GC from LC amenable compounds in one sample procedure utilising a chromatographic separation reduces the number of methods, while avoiding loss of analytes through aliquoting of the extract. Furthermore, one method can target both PBDEs and emerging HFRs with diverse physicochemical properties. Both methods described in this manuscript include a purification step to remove lipids and a fractionation step to isolate the GC from the LC amenable compounds. The list of HFRs included in this study comprised 33 BFRs, including 14 PBDE congeners, 19 other brominated compounds, and four chlorinated flame retardants. Two methods were compared: one used an HPLC instrument for purification by GPC followed by Partisil fractionation on an amino-cyano normal phase and the other method was a more traditional method based on H_2_SO_4_ treatment followed by fractionation by silica column chromatography. The analysis of PBDEs and other emerging HFRs was determined by GC-tandem mass spectrometry (MS/MS), and the analysis of HBCDD isomers and TBBPA was analysed by LC-MS/MS.

## Materials and methods

### Standard solutions

The native and isotope-labelled standard solutions were supplied by Accustandard (New Haven, USA) and Wellington Laboratories (Ontario, Canada). The list of the target HFRs with their structure and basic physicochemical constants is compiled in Table S1. They included 14 BDE congeners (17, 28, 47, 66, 71, 85, 99, 100, 138, 153, 154, 183, 190, 209), 19 brominated compounds (2,4,6-tribromophenol (TBP), 2,4,6-tribromophenyl allyl ether (TBP-AE), pentabromotoluene (PBT), pentabromoethylbenzene (PBEB), 2-ethylhexyl 2,3,4,5-tetrabromobenzoate (EHTBB), pentabromobenzene (PBB), hexabromobenzene (HBB), pentabromobenzyl acrylate (PBB-Acr), bis(2-ethylhexyl) tetrabromophthalate (BEH-TEBP), tetrabromobisphenol A (TBBPA), tetrabromobisphenol A bismethyl ether (TBBPA-BME), 1,2-bis(2,4,6-tribromophenoxy)ethane (BTBPE), decabromodiphenylethane (DBDPE), 4-(1,2-dibromoethyl)-1,2-dibromocyclohexane (DBE-DBCH-α and DBE-DBCH-β) isomers, hexachlorocyclopentadienyl dibromocyclooctane (DBHCTD), hexabromocyclododecane (α-HBCDD, β-HBCDD, and γ-HBCDD) isomers), and four chlorinated flame retardants (dechlorane 602 (Dec 602), dechlorane 603 (Dec 603), and dechlorane plus syn and anti (DDC-CO) isomers). Bisphenol A, supplied by Accustandard, was also included in the analyte list. Although bisphenol A is not a flame retardant, it is a chemical additive in many consumer products.

The GC and LC calibration and surrogate standards were prepared by serial dilutions in isooctane and methanol, respectively. The surrogate solution for GC contained labelled ^13^C_12_-BDEs (77/118/183) at 1 μg ml^−1^. The surrogate solution for LC included labelled ^13^C_12_-TBBPA, d_18_-β-HBCDD and d_18_-γ-HBCDD at 1 μg ml^−1^. Before the GC analyses, 10 μl of a GC internal standard solution of Tetrachloro-m-xylene (TCMX) at 1 μg ml^−1^ in isooctane was spiked into the vials. For the LC analyses, 10 μl of a solution of d_18-_α-HBCDD in methanol at 1 μg ml^−1^ was spiked into the LC vials. All solutions were stored at 4 °C in darkness.

A standard solution of F-BDE 208 and a mix of PAHs standards purchased from AccuStandard were used to monitor the Partisil fractionation on a polar amino-cyano silica column.

A GPC calibration mix was prepared with olive oil (Carrefour), the synthetic organochlorine pesticide methoxychlor (Chem Service), and perylene (AccuStandard).

### Reagents and solvents

Methanol, dichloromethane (DCM), n-hexane, isooctane, and acetone for residue analyses and HPLC-grade methanol and HPLC-grade ammonium acetate were purchased from Fisher Scientific (France). Sulphuric acid (98%) and anhydrous sodium sulphate were obtained from MERCK (France). Ultra-pure water was produced on-site using a Millipore Milli-Q system (specific resistivity of 18.2 MΩ cm, 25 °C). Silica columns (2 g, 45 μm particle size, and pore size of 60 Å) in glass cartridges were supplied by Macherey Nagel (France).

Sodium sulphate (Na_2_SO_4_), glass wool, and glassware were combusted at 450 °C for at least 6 h.

### Samples

Four grams (dried weight, dw) of a mussel reference material (IAEA-432, North Sea) with native HFRs lower than the method detection limits (Tables S7 and S8) was spiked with the target HFRs at 5 ng g^−1^ dw, or 25 ng g^−1^ dw for BDE 209 and DBDPE. Other IAEA biota reference materials: IAEA-415 and IAEA-442 (fish from North Sea), IAEA-435 (Mediterranean fish), IAEA-437 (Mediterranean mussel), IAEA-406 (fish from aquaculture), IAEA-451 (clam from New Caledonia), and mussel CRM of NIST 2974a were also analysed.

### Sample preparation

**Fig. 1:**
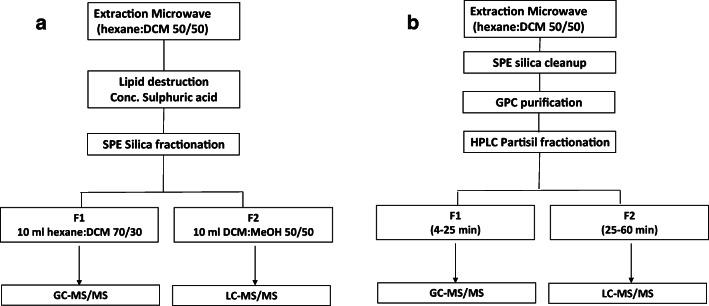
Sample treatment procedures. **a** Traditional method based on H_2_SO_4_ clean-up of the extracts followed by fractionation by silica column chromatography (H_2_SO_4_-silica). **b** A more automated method with SPE pre-clean-up step, GPC clean-up followed by a Partisil fractionation using polar amino-cyano normal phase chromatography (GPC-Partisil)

As a short overview of the sample preparation, all samples were extracted using microwave-assisted extraction (MAE). Two different purification and fractionation procedures were developed and compared. The first procedure (Fig. 1a) is a more traditional method, and included concentrated sulphuric acid treatment to remove lipids followed by a solid-phase extraction (SPE) silica column fractionation (see below the “Sulphuric acid treatment and SPE silica fractionation (H_2_SO_4_-silica) procedure” section for this procedure). The second method (Fig. 1b) included a preliminary SPE silica column clean-up step, followed by further removal of lipids by GPC, and Partisil fractionation on a polar amino-cyano normal phase (see below the “High-performance analytical GPC and Partisil fractionation (GPC-Partisil) procedure” section for this procedure).

#### Extraction step

A known amount of homogenised dried biota was weighed in a CEM GreenChem glass vessel (typically 2–4 g). Twenty microliter of the GC and LC surrogate solutions and 40 mL of the extraction solvent (hexane:DCM 50/50 v:v) were added to each sample. The samples were microwave extracted using a Mars Xpress extractor (CEM, France) at 1200 W. The temperature reached 115 °C in 10 min, and then remained at 115 °C for 20 min before cooling.

The extracts were transferred to Buchi Syncore evaporation flasks via glass wool plugged funnels, rinsing the vessels and funnels with DCM. The solvent was evaporated in a Buchi Syncore evaporator (France) (up to 12 samples at once) using an appropriate hexane:DCM evaporation program. The concentrated extracts were transferred to graduated glass tubes and the volume was adjusted to 1 mL with DCM.

#### Lipid content

The lipid content was calculated gravimetrically using an Ultra-micro balance Mettler Toledo UMX2 (France) with a precision of ± 0.1 μg. To determine the lipid content, a 10 μl aliquot of the 1 mL extract was evaporated to dryness and the mass was recorded. If the extract contained less than 2 μg of lipids, the extract was concentrated prior to the lipid removal step.

#### Sulphuric acid treatment and SPE silica fractionation (H_2_SO_4_-silica) procedure

##### Lipid removal with sulphuric acid

The 1 mL extracts were transferred to 10-mL Teflon tubes for liquid-liquid extraction (LLE). The glass tubes were rinsed twice with 1 mL of DCM and the rinsate was transferred to the Teflon tube. Approximately 1–2 mL of concentrated sulphuric acid (98%) was added to the extracts using a Pasteur pipette; the tubes were capped, shaken by hand, and centrifuged (5000 rpm, 5 min). After centrifugation, 2 layers were formed: the bottom layer contained H_2_SO_4_ and the lipid degradation products, and the top layer contained the HFRs in DCM. The acid layer was carefully removed using a pasteur pipette and the process was repeated at least 2 more times until the DCM layer appeared nearly colourless. After centrifugation, the DCM layer was carefully transferred to a clean glass test tube and evaporated to < 500 μL.

##### SPE silica column fractionation

The sulphuric acid cleaned extracts were fractionated by SPE silica column chromatography (2 g), topped with approximately 2 mm of Na_2_SO_4_ (~ 0.5 g) to remove residual sulphuric acid. The silica column was conditioned with 10 mL of hexane before loading the sample. The first fraction was eluted with 10 mL hexane:DCM (70/30) and contained all the GC amenable compounds except TBBPA-BME and PBB-Acr. The second fraction was eluted with 10 mL of DCM:methanol (50/50) and contained all the LC amenable compounds and TBBPA-BME and PBB-Acr.

#### High-performance analytical GPC and Partisil fractionation (GPC-Partisil) procedure

##### SPE silica column pre-clean-up

Initially, the 1 ml concentrated extract was loaded onto a SPE silica column (2 g) preconditioned with 10 mL DCM to remove lipids prior to the GPC clean-up. The extract was eluted with 10 mL DCM.

##### High-performance analytical GPC clean-up

**Fig. 2:**
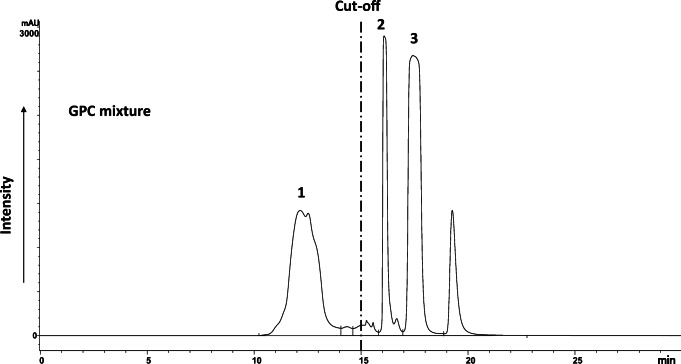
Representative GPC chromatogram by PDA (254 nm) of a mixture of olive oil (1), methoxychlor (2), and perylene (3) used to calibrate the fractionation of the lipids from the target compounds. The cut-off for lipid removal was set at 15 min

The extract was concentrated to ~ 950 μl under a gentle stream of nitrogen and transferred to a high-recovery vial (1 ml with 9 μL residual volume, Waters Part Number 186000385C) prior to GPC clean-up. GPC was performed on a Waters Alliance HPLC system equipped with an e2695 XC Separations Module (featuring a quaternary LC pump and a thermostated column compartment), a 2500 μl syringe with sample loop of 2 mL coupled to a 2998 Photodiode Array (PDA) detector, and a Waters WFC III fraction collector. GPC was performed by injecting the entire sample (~ 950 μL) onto two Agilent PLGel columns in series (cross-linked polystyrene divinylbenzene, particle diameter 5 μm, pore size 50 Å, 300 × 7.5 mm) with DCM as the mobile phase. The GPC injection program is summarised in Table S2. The good performance of the system was verified using a calibration mix containing olive oil, the synthetic organochlorine pesticide methoxychlor, and the polycyclic aromatic hydrocarbon (PAH) perylene (Fig. 2). The HFR fraction eluted from 15 to 30 min. The purified extract was evaporated to approximately 1 mL and transferred to a new high-recovery vial using isooctane as a rinse solvent. The extract was evaporated to ~ 400 μL.

##### HPLC fractionation on Partisil column

The GPC extract was fractionated for GC and LC analysis on the Waters Alliance HPLC system using a polar amino-cyano normal phase Partisil PAC5 column (Interchim, France, particle diameter 5 μm, 80 Å, 250 × 4.0 mm) with a polar amino-cyano normal phase guard column (Interchim, France, particle diameter 5 μm, 80 Å, 20 × 4.0 mm). The entire extract (400 μL) was injected onto the Partisil column. The chromatographic gradient covered a broad range of polarity solvents including hexane, DCM, and methanol as described in Tables S3 and S4.

**Fig. 3:**
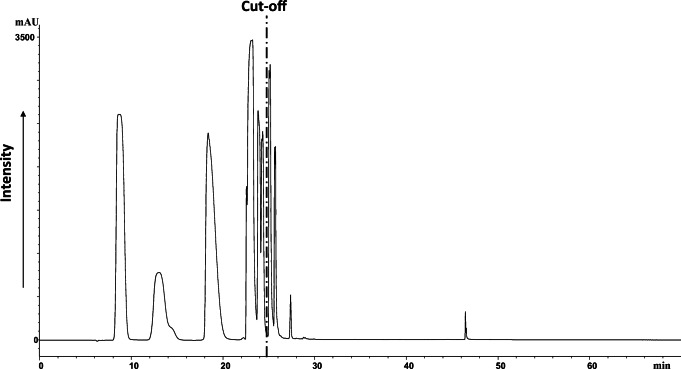
Representative Partisil-PDA chromatogram (254 nm) of PAHs standards used to calibrate the fractionation of GC and LC amenable compounds. The cut-off for GC/LC fractionation is set at 25 min

The good performance of the system was verified in each batch using a calibration mix of hydrocarbons. The elution of PAHs was monitored using PDA detection absorbance set at 254 and 235 nm (Fig. 3). Another standard solution of F-BDE 208, which eluted around 24 min, was used to assess the elution of the higher molecular weight PBDEs (> 700 Da). The GC amenable compounds eluted in the hexane:DCM fraction between 4 and 25 min and the LC amenable compounds eluted in the DCM:methanol fraction between 25 and 60 min.

### Instrumental analysis

#### GC-MS/MS

The GC fraction extract was evaporated to 200 μL using isooctane as a guard solvent and the GC internal standard, TCMX, was added (10 μL of a 1 µg mL^-1^ solution in isooctane) prior to analysis.

GC analysis was performed using an Agilent GC 7890B system coupled to a 7000C triple quadrupole mass spectrometer equipped with an electron impact source (70 eV, 230 °C) and operated in multiple reaction monitoring (MRM) mode. Chromatographic separation was achieved on an Agilent DB-5MS column (15 m × 0.25 mm, film thickness 0.1 μm) using He as a carrier gas, at a flow rate of 1.7 mL min^-1^. Sample (injection volume 1 μL) was introduced using a programmable temperature vaporising inlet (PTV) at 85 °C (held for 0.1 min), increased to 270 °C at 120 °C min^-1^ (held for 0.5 min). A 150 μL multi-baffle liner was used. The inlet was purged of non-volatile residues at 60 mL min^-1^ after 4 min by opening the vent valve. The oven initial temperature was set at 60 °C, held for 2 min, increasing to 120 °C at 20 °C min^-1^ and to 310 °C at 18 °C min^-1^, with a final hold time of 20 min. The transfer line was kept at 310 °C.

The monitored MRM transitions, concentration range of calibrants, correlation coefficients in linearity tests, and the instrumental limit of detection (ILOD) are given in Table S5.

#### LC-MS/MS

The LC fraction was evaporated to dryness and reconstituted in 100 μL of methanol. The LC internal standard, d_18_-α-HBCDD, (10 μL of a 1 µg mL^-1^ solution) was added to the extract. Prior to injection, a 20 μL aliquot of the extract was mixed with 20 μL of milli-Q water.

Extracts were analysed by electrospray ionisation (ESI) using a Waters H-Class LC system coupled to a Xevo TQD triple quadrupole mass spectrometer as previously described in Aminot et al. (2020). Briefly, chromatographic separation was achieved on a Waters BEH C18 column (particle size 1.7 μm, 100 × 2.1 mm) using as mobile phases milli-Q water and HPLC-grade methanol, both containing 0.2 mM of HPLC-grade ammonium acetate.

The MS was operated in MRM mode, with a dwell time of 24 ms. The MS parameters, concentration range of calibrants, correlation coefficients in linearity tests, and ILOD are given in Table S6.

### Quality assurance and control

Procedural reagent blanks (*n* = 3), solvent spike recovery samples (*n* = 3), blank matrix samples (*n* = 3), and matrix spike recovery samples (*n* = 3) were analysed for each procedure. Repeated experiments were performed on different days and by two different operators to calculate recoveries, repeatability (intra-day precision), and reproducibility (inter-day precision) (Table S7).

Method detection limits (MDLs) were set as 3 times the standard deviation of the target analytes detected in the procedural reagent blanks. For compounds not detected in the blanks, MDLs were based on a signal-to noise ratio of three. Matrix blank levels of target compounds constituted less than 1% of the respective spike levels in matrix sample. All matrix spike recoveries values were blank matrix corrected by subtracting blank matrix concentrations from matrix spike sample concentrations.

The absolute extraction recovery of target analytes and surrogate compounds was performed using the internal standards (TCMX or d_18_-α-HBCDD). Correlation coefficients in linearity tests were all > 0.99 (Table S5 and S6). The mussel standard reference material (SRM) NIST 2974a was used to evaluate the accuracy of the procedures for some PBDEs and HBCDD isomers (Table S8). There is no reference material that contains all the target analytes. Our agreement with the NIST 2974a certified values is satisfactory, with recovery values ranging from 70 to 125%.

## Results and discussion

### Sulphuric acid treatment

Sulphuric acid treatment is one of the clean-up procedures frequently used to remove lipid content in biota tissue samples when analysing for legacy persistent organic pollutants (POPs) and HFR contaminants (Covaci et al. 2007). PCBs, PBDEs, HBCDDs, and TBBPA are resistant to sulphuric acid treatment (Covaci et al. 2003; Nácher-Mestre et al. 2010; Sporring and Björklund 2004; Inthavong et al. 2017) but the stability of the emerging HFRs is still under controversy and was assessed in this procedure.

Most of the recoveries of the GC amenable HFRs treated directly with concentrated sulphuric acid fell between 60 and 120% with relative standard deviation (RSD) < 20% (Fig. S1) These results are in accordance with the EU guidance document for pesticide residues analysis in food, where the acceptable mean recoveries are in the range of 70–120% and the associated repeatability of relative standard deviation RSD < 20% (European Commission 2017). TBP-AE had a recovery of 58 ± 10% (as mean of 3 replicates ± standard deviation) in pure solvent, and a 0% recovery when spiked into biota (see the “Comparison of the two sample treatment procedures” section). This observation agreed with other authors who have also reported complete degradation of TBP-AE on acidified silica (Geens et al. 2010; Yang et al. 2012).

Other studies showed that several HFR compounds were sensitive to and/or degraded by sulphuric acid. For instance, some studies have shown low recovery of BTBPE on acidified silica due to the partial hydrolysis of the oxygen of the phenoxy group (López et al. 2011), whereas other studies showed acceptable recoveries with acidified silica (Yang et al. 2012; Van den Eede et al. 2012) and concentrated sulphuric acid (Ilyas et al. 2011). BEH-TEBP, EHTBB, and PBB-Acr were also reported to degrade in acidic conditions (Van den Eede et al. 2012; Yang et al. 2012; Carlsson et al. 2018) and yet, our recoveries are within the acceptable range. Limiting the contact time between sulphuric acid and the extract could account for our higher recoveries in standard solutions, as we also observed losses after repeated LLE for biota matrices (the “Comparison of the two sample treatment procedures” section).

The recoveries of the LC amenable HFRs were satisfactory for the HBCDD isomers and TBBPA, and consistent with other reported data (Han et al. 2017). Bisphenol A was completely degraded by sulphuric acid and the recovery of TBP not reproducible.

### SPE fractionation development

Florisil and silica sorbents were tested for the SPE fractionation step using spike and recovery experiments to test different elution volumes and solvent ratios. Silica retained more compounds and separated the GC and LC amenable HFRs better than Florisil; therefore, it was selected as the sorbent. The first fraction, which contained GC amenable HFRs, was eluted with 10 mL hexane:DCM (70/30) and the second fraction, which contained the LC amenable HFRs, was eluted with 10 mL of DCM:Methanol (50/50). The recoveries of the GC amenable HFRs (as mean of 3 replicates ± standard deviation) are shown in Fig. S2. Under these conditions, the first fraction contained all the GC amenable HFRs except BEH-TEBP, which was not recovered in either fraction; EHTBB, which splitted among both fractions; and PBB-Acr and TBBPA-BME, which eluted in the second fraction. The LC amenable HFRs were recovered in the second fraction with acceptable recoveries of 60–105%.

### GPC clean-up

Two PLgel GPC columns were used in series to remove higher molecular weight lipids, such as triclycerides or phospholipids. The GPC columns have an exclusion limit around 5000 Da; therefore, the lipids are excluded from the pores and elute before the target analytes. The PLgel analytical column requires less solvent and has a faster run time than a 25 mm preparative GPC column, while still offering high-resolution chromatographic separation. Unfortunately, the total amount of lipid injected on to the column must be less than 100 mg; otherwise, breakthrough might occur. Some biota samples can contain up to 160 mg g^−1^ lipid dw; therefore, prior to the GPC clean-up step, a silica column purification step was added, which removed 39 ± 3% of the initial lipids.

The GPC fraction collected between 15 and 30 min contained the target HFRs (Fig. 2). Even after GPC clean-up, 2–5% of the initial lipids remained, indicating the need to apply another clean-up step such as normal phase column chromatography.

### Partisil fractionation development

A Partisil column was selected for fractionation after GPC clean-up because it is amenable to a broad range of mobile phase polarities and allows for a variety of separation mechanisms which are necessary to reproducibly separate the GC and LC amenable HFRs. The Partisil 5 PAC column is made of a polar amino-cyano (NH_2_-CN) (2:1 ratio) silica–bonded phase, allowing a fast stabilisation of the column even with a broad range of solvent polarities. Furthermore, the NH_2_-CN bonded group permits adsorption, weak-anion exchange, and reversed-phase mechanisms to provide the best separation of compounds of interest. Fractionation optimisation was performed with the target HFRs to determine the elution pattern of GC and LC fractions. The tests followed the same experimental protocol as described in previous work (Tolosa and de Mora 2004), where the elution started with 100% hexane, a non-polar solvent, and finished with a polar mixture of DCM and methanol. The number of fractions, the solvent ratios, and elution times were optimised for HFR separation. The window for high molecular weight PBDEs in the GC fraction was determined by monitoring the elution of F-BDE 208 with PDA (235 nm). Based on F-BDE 208 retention time, the fraction that contained the GC amenable HFRs was collected between 4 and 25 min and the fraction that contained the LC amenable HFRs was collected between 25 and 60 min. The recoveries of all HFRs were satisfactory in the respective fractions, except TBBPA-BME, which was found in both fractions (80% in the GC fraction and 20% in the LC fraction) and bisphenol A, which was lost due to its poor solubility in isooctane, the sample solvent.

### Comparison of the two sample treatment procedures

Lipid removal is a critical step when analysing organic contaminants in biota by MS/MS. A mussel tissue (IAEA-432), which contained 63 mg g^−1^ of lipid dw, was used to assess the two sample treatment methods for lipid removal efficiency. The sulphuric acid treatment removed 99.5 ± 0.1% of lipids from the mussel tissue while the SPE silica only treatment removed 39 ± 3% and the GPC method removed 97 ± 3% of total lipids. If the sample contained more than 100 mg of lipid, then the silica column was necessary to prevent breakthrough or overloading of the GPC. This procedure was followed by additional lipid removal by Partisil normal phase fractionation to reduce the matrix load even further, indicating potential irreversible adsorption on the column/precolumn. Overall, the GPC-Partisil procedure removed 99.9 ± 0.1% of the lipids (Fig. S3).

**Fig. 4:**
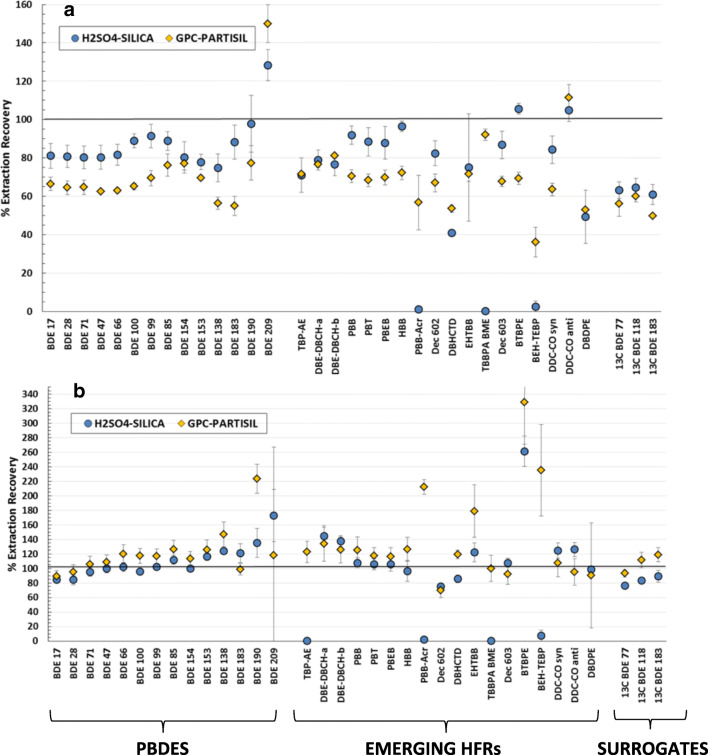
Absolute extraction recovery yields of the GC amenable HFRs and surrogate standards in **a** spiked solvent samples and **b** in spiked mussel samples (IAEA-432) by (blue) H_2_SO_4_ treatment of the extracts followed by silica column fractionation and (yellow) GPC purification followed by Partisil fractionation. Absolute recoveries were quantified using TCMX as the internal standard, and TBBPA-BME and PBB-Arc were recovered in the LC fraction. The values are the mean of 3 replicates and the error bars represent the ± standard deviation

The absolute recovery for the individual GC amenable compounds was calculated using TCMX as the internal standard. Figure 4 shows the absolute extraction recovery, as the mean of 3 replicates ± standard deviation, of the target GC amenable HFRs and surrogate compounds (^13^C_12_-BDEs 77/118/183) in spiked solvent (4a) and in IAEA-432 spiked mussel samples (4b), using the two different sample treatment procedures: (i) H_2_SO_4_ treatment of the extracts followed by SPE silica column fractionation (blue circles) and (ii) GPC purification followed by Partisil fractionation (yellow diamonds).

The majority of the PBDE compounds, including the surrogates, had absolute recoveries between 60 and 120% with relative standard deviation (RSD) < 15% for both treatment procedures in spiked solvent and spiked mussel samples. Only the higher brominated congeners, such as BDE 190 and BDE 209, exhibited particularly high recoveries and variability in both treatments, reflecting the known problems associated with the instrumental analyses and their potential degradation at higher temperatures (de Boer et al. 2001).

Concerning the emerging GC amenable HFRs, both procedures provided satisfactory absolute recoveries for DBE-DBCH isomers, PBB, PBT, PBEB, HBB, Dec-602, DBHCTD, Dec-603, and Dec Plus isomers. The absolute recovery for DBDPE was similar to the ^13^C_12_-BDEs surrogates (60–120%) but had high variability for the spiked matrix and GPC-Partisil procedure (57% RSD). These inconsistencies reflect the similar instrumental problems observed with BDE 209. On the other hand, TBP-AE, PBB-Acr, TBBPA-BME, and BEH-TEBP were not recovered on the H_2_SO_4_-silica treatment procedure: TBP-AE was degraded by sulphuric acid in the spiked matrix; PBB-Acr and TBBPA-BME eluted in the LC fraction; and BEH-TEBP was lost on the silica column. The higher recoveries of DBE-DBCH (α+β), EHTBB, BTBPE, BEH-TEBP, and PBB-Acr in the spiked mussel matrix could not be attributed to the presence of native contaminant in the mussel but could either be related to (1) matrix enhancement caused by co-eluting components, which altered the ionisation of target analytes (Panuwet et al. 2016), or (2) matrix suppression of the associated surrogate(s). Nevertheless, EHTBB, BTBPE, BEH-TEBP, and PBB-Acr exhibit relatively high instrumental detection limits (IDLs) (Table S5) in the GC-MS/MS, leading to higher relative variability in the spiked samples (RSD > 15%). Our results align with the reported matrix effects for DBE-DBCH isomers and EHTBB in river surface waters analysed by GC-MS/MS (Gustavsson et al. 2017).

**Fig. 5:**
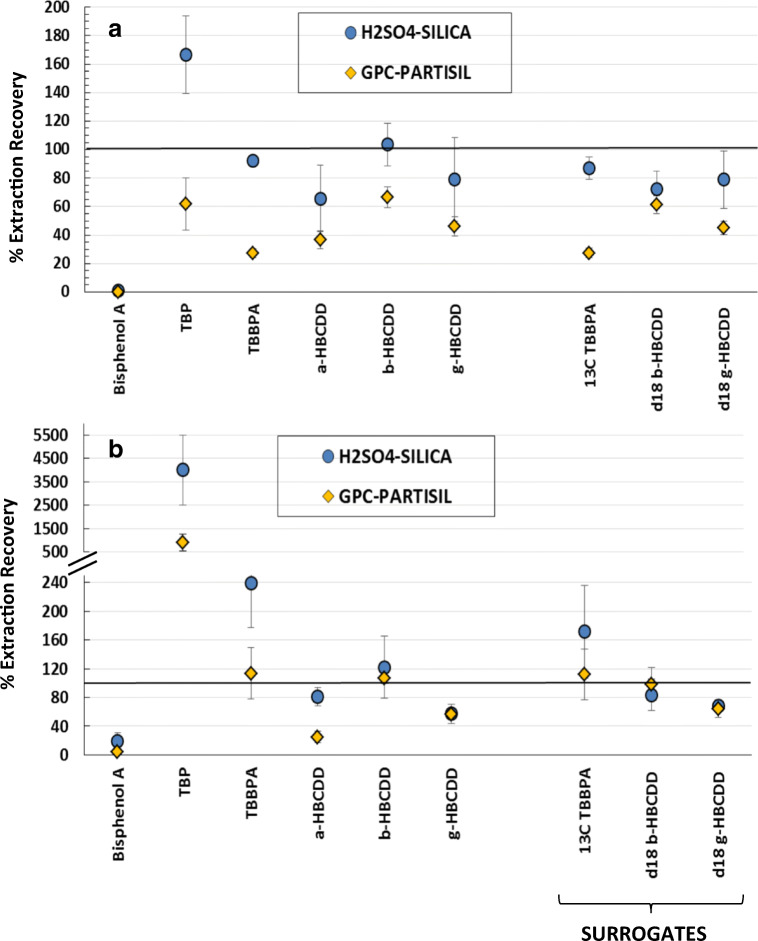
Absolute extraction recovery of the LC amenable HFRs and surrogates standards in **a** spiked solvent and **b** in spiked mussel samples (IAEA-432) by (blue) H_2_SO_4_ treatment of the extracts followed by silica column chromatography and (yellow) GPC purification followed by Partisil fractionation. Absolute recoveries were quantified using d_18_-α-HBCDD as the internal standard. The values are the mean of 3 replicates and the error bars represent the ± standard deviation

**Fig. 6:**
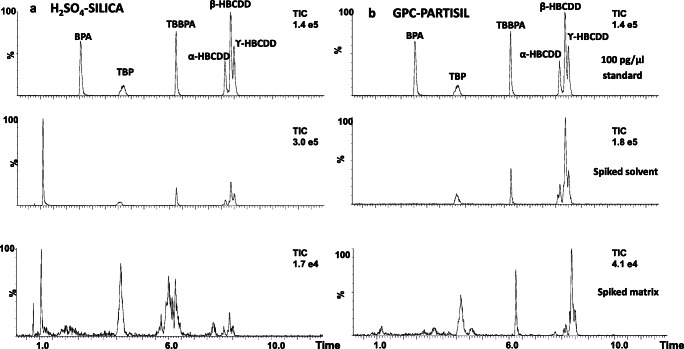
LC-MS/MS Total ion chromatogram (TIC) comparing H_2_SO_4_-silica (**a**) vs GPC-Partisil (**b**) with a standard mix (top), and solvent spiked (middle), and spiked mussel matrix extracts (bottom)

The absolute extraction recoveries of the target LC amenable HFRs and surrogate compounds (^13^C_12_-TBBPA, d_18_-β-HBCDD, and d_18_-γ-HBCDD) for both procedures in spiked solvent (*n* = 3) and spiked IAEA-432 mussel samples (*n* = 3) are shown in Fig. 5, as the mean of 3 replicates ± standard deviation. We found differences between the isomers of HBCDD, which indicated isomer-specific losses in both procedures. This illustrates the need to use individual isotope-labelled standards for accurate quantification. The recovery of TBBPA differed between the spiked solvent (27 ± 3% by GPC-Partisil) and the spiked mussel matrix (240 ± 63% by H_2_SO_4_-silica), and between both treatment procedures. Similar behaviour was observed for its surrogate (^13^C_12_-TBBPA) which warrants its accurate quantification. Bisphenol A was not recovered by either treatment because it has limited solubility in isooctane, the solvent used in the HPLC fractionation, and was degraded by the sulphuric acid treatment. Absolute recoveries of TBP and TBBPA were high in the H_2_SO_4_ treatment, which revealed that the matrix effect was more significant with H_2_SO_4_-silica treatment procedure. This was also confirmed on the chromatographic profile (Fig. 6) on which the peak shape was distorted after H_2_SO_4_ treatment in spiked mussel matrix (Fig. 6a bottom), but not in the spiked solvent extracts (Fig. 6a middle). Again, the abnormally high recoveries of TBBPA (239 ± 63%) and TBP (4007 ± 1502%) in the mussel matrix are likely related to matrix enhancement, as TBBPA was absent in the mussel matrix and TBP was relatively low (8.8 ng g^−1^ dw). In addition, signal suppression was observed on the internal standard, d_18_-α-HBCDD. In this aspect, the use of isotope-labelled standards is the most effective way to correct for matrix effects. However, isotope-labelled standards are costly and sometimes unavailable. Matrix-matched calibration offers also an alternative approach to compensate for the matrix effects as long as the composition of the matrix between samples remains similar (Gustavsson et al. 2017). Nevertheless, many other significant drawbacks of the matrix-matched calibration arise including finding appropriate free-analytes matrices, increasing the sample workload, and maintaining the instruments more frequently resulting from the increase of matrix material injections.

For both procedures, the method detection limits (MDL) for PBDEs (except BDE 209) and HBCDD isomers ranged from 0.01 to 0.12 ng g^−1^ dw (Table S7) using a 4 g sample. The MDLs for PBDEs and HBCDD isomers fall within the recommendations of the EU Commission (European Commission 2014) which endorses analytical methods with a limit of quantification (LOQ) below 0.01 ng g^−1^ wet weight (ww) in fish and other seafood (~ 0.05 ng g^−1^ dw using a conversion ratio from ww to dw of 5 (EPA 2011)). The MDL for the brominated phenols, TBP, and TBPPA, was 0.01 ng g^−1^ dw in both methods; this value also falls below the EU Commission’s recommended values of 0.1 ng g^−1^ ww (0.5 ng g^−1^ dw). For the remaining HFRs, LOQs were below the guideline of 1 ng g^−1^ ww (European Commission 2014).

### Application to biota reference samples

The GPC-Partisil procedure was used to assess the level of the HFRs in IAEA biota reference materials, which are under preparation or certified for other elements (e.g. trace elements, radionuclides, or other POPs). Only PBDEs, TBP, α-HBCDD, and DBE-DBCH isomers were detected above the MDL in seven IAEA reference materials (Table S8). The concentration of PBDEs ranged from 0.72 to 26.7 ng g^−1^ dw in the marine biota with BDE-47, BDE-100, and BDE-154 as the predominant BDEs in the fish material. All PBDE detections exceeded the environmental quality standard (EQS) set by the EU Commission of 0.009 ng g^−1^ ww (~ 0.042 ng g^−1^ dw) (European Commission 2013). The EQS refers to protection limit for “human health” and it is based on the sum of BDE 28, 47, 99, 100, 153, and 154. Recently, PBDE concentrations in marine biota from Europe have significantly exceeded the low EQS (0.030–5.6 ng g^−1^ ww; Zhihua et al. 2018; Munschy et al. 2015; Airaksinen et al. 2015) causing controversy over the limit set by the EU Commission (Eljarrat and Barceló 2018). Environment Canada established a higher EQS for PBDEs in the Federal Environmental Quality Guidelines (FEQGs), which is more relevant to environmental concentrations. The lowest value is assigned to the pentaBDE homologues, with individual values of 1 ng g^−1^ ww for BDE 99 and BDE 100, and a total tetraBDE of 88 ng g^−1^ ww in fish tissue (FEQG 2013). Given these established EQS limits, the production of natural reference materials for PBDEs in marine biota should be achievable.

Among the LC analytes, TBBPA was less than MDL in all reference samples analysed, whereas TBP was found at relatively high concentrations in bivalve samples ranging from 6.9 to 27.3 ng g^−1^ dw, and much lower values in fish samples from 0.03 to 2.7 ng g^−1^ dw. Higher concentrations of TBP were also observed in mussel samples compared with fishes, which could be due to higher elimination rates of TBP in fishes (Aznar-Alemany et al. 2017).

Of the HBCDD isomers, only α-HBCDD was detected in one Mediterranean fish (IAEA-435) and one Mediterranean mussel (IAEA-437) with similar concentration levels of 0.49 ng g^−1^ dw (0.098 ng g^−1^ ww). These values agree with previously reported HBCDD concentrations in shellfish from Europe (0.03–0.4 ng g^−1^ ww, Munschy et al. 2015), and are well below the biota-EQS of 167 ng g^−1^ ww in fish (sum of *α*, *β*, and γ-HBCDD) (European Commission 2013).

Among the emerging GC amenable HFRs, only DBE-DBCH isomers were detected in all reference materials at concentrations levels ranging from 1.2 to 88.2 ng g^−1^ dw (0.24 to 17.7 ng g^−1^ ww). These concentrations are up to 150 times higher than fish caught in Sweden (Sahlström et al. 2015), about 10 times higher than from the UK (Tao et al. 2017) and Hong Kong (Ruan et al. 2018). DBE-DBCH-α was the dominant isomer in all reference materials, with a ratio of *α*/*β* of 1.41 ± 0.06. Similar isomeric ratios were reported in the technical mixture (Arsenault et al. 2008), in Swedish seafood samples (Sahlström et al. 2015), and in cetaceans from South China Sea (Ruan et al. 2018). In contrast, DBE-DBCH-β was the predominant isomer in seafood from the UK (Tao et al. 2017) and artic beluga from Canada (Tomy et al. 2008), suggesting that the uptake and metabolism of DBE-DBCH isomers might be species dependent (Tao et al. 2017). The extremely high levels of DBE-DBCH isomers found in the fish reference materials from North Sea (IAEA-442 and IAEA-415) suggest that these isomers are either widespread in the area or these samples were contaminated during preparation of the bulk reference materials (freeze-drying, sieving, homogenisation, bottling). Further monitoring studies in the marine environment should be envisaged to confirm the occurrence and impact of these emerging flame retardants.

## Conclusions and recommendations

In conclusion, the GPC-Partisil procedure yielded a high recovery for all target HFRs in biota samples including those more labile that might be degraded by sulphuric acid. The method detection limits comply with the recommendations of the EU commission. The GPC-Partisil procedure is more automated, thus reduces bench work and is more reproducible than the H_2_SO_4_-silica procedure. Nevertheless, the procedure requires a HPLC instrument which is expensive and requires trained personnel.

In this study, we provided evidence that (1) a non-destructive clean-up procedure is needed for several of the novel HFR (e.g. TBP-AE) and (2) fractionation with SPE silica column is not selective enough to separate GC from LC amenable compounds (e.g. TBBPA-BME, PBB-Acr, and BEH-TEBP). The combination of silica column pre-clean-up followed by GPC and Partisil fractionation could be optimised for lipid removal and fractionation of GC and LC amenable HFRs in biota. Co-eluting matrix components were responsible for ion suppression/enhancement during ionisation MS; however, the use of isotope-labelled standards or matrix-matched calibration curves compensates for matrix effects. In addition, we need reference standards (labelled and native) and appropriate matrix certified reference materials (CRMs) to validate the analytical methods that will be used in monitoring emerging and historical environmental contaminants globally. The application of this method to marine biota reference materials revealed high concentrations of the DBE-DBCH isomers in fish samples, which deserves further assessment of these emerging contaminants in the marine environment.

## Electronic supplementary material

ESM 1 (DOCX 404 kb)

## Data Availability

Most of the data generated or analysed during this study are included in this published article and its supplementary information files. All data generated during the current study are available from the corresponding author on reasonable request.
