# Supplementary material for: A comprehensive evaluation of two sample treatment procedures for the determination of emerging and historical halogenated flame retardants in biota
Source: Environ Sci Pollut Res Int. 2020 Oct 7;28(42):59345–57. doi: 10.1007/s11356-020-10966-y (PMC8542007; doi:10.1007/s11356-020-10966-y)
Supplement: Supplementary file 1 — (DOCX 404 kb) [file 11356_2020_10966_MOESM1_ESM.docx]

**Electronic Supporting Information (ESI)**

**A comprehensive evaluation of two sample treatment procedures for the determination of emerging and historical halogenated flame retardants in biota**

**I. Tolosa^1^*, D. Huertas^1^, S. Choyke^1^, S. Sander^1^, Y. Aminot^1,2,^**

1. IAEA Environment Laboratories, 4a Quai Antoine 1er, 98000 Monaco
2. current address: IFREMER, Laboratory of Biogeochemistry of Organic Contaminants, Rue de l’Ile d’Yeu, BP 21105, 44311 Nantes Cedex 3, France

*Corresponding author: [I.Tolosa@iaea.org](mailto:I.Tolosa@iaea.org)

Table S1. List of halogenated flame retardants investigated in this study. Adapted from Bergman et al., 2012.

| CAS number | acronym | Structure | Common and trade names | MW | Log Kow | Koc | pKa | Vapor pressure (Pa) |
| --- | --- | --- | --- | --- | --- | --- | --- | --- |
| 118-79-6 | TBP | 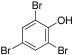 | 2,4,6-Tribromophenol | 330.8 | 4.4 | pH-dep. | 6.34 ± 0.23 | 2.00E-01 |
| 3278-89-5 | TBP-AE | 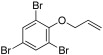 | 2,4,6-Tribromophenyl allyl ether | 370.8 | 5.04 | 13,100 | na | 2.40E-02 |
| 87-83-2 | PBT | 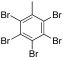 | Pentabromotoluene | 486.62 | 6.25 | 60,200 | na | 6.00E-04 |
| 85-22-3 | PBEB | 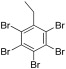 | Pentabromoethylbenzene | 500.65 | 6.76 | 1.14E + 05 | na | 1.56E-04 |
| 183658-27-7 | EHTBB | 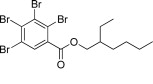 | 2-Ethylhexyl 2,3,4,5-tetrabromobenzoate | 549.92 | 7.73 | 3.82E + 05 | na | 3.71E-07 |
| 608-90-2 | PBB | 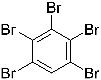 | Pentabromobenzene | 472.59 | 5.4 |  | 6.5e-4 |  |
| 87-82-1 | HBB | 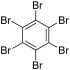 | Hexabromobenzene | 551.49 | 6.11 | 50,300 | na | 1.14E-04 |
| 59447-55-1 | PBB-Acr | 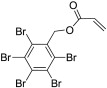 | Pentabromobenzyl acrylate | 556.67 | 5.6 | 26,500 | na | 3.64E-07 |
| 26040-51-7 | BEH-TEBP | 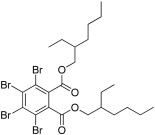 | Bis(2-ethylhexyl) tetrabromophthalate | 706.14 | 9.34 | 2.88E + 06 | na | 1.55E-11 |
| 79-94-7 | TBBPA | 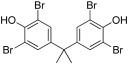 | Tetrabromobisphenol A | 543.87 | 9.69 | 4.47E + 06 | 7.5/8.5 ± 0.1 | 1.88E-05 |
| 37853-61-5 | TBBPA-BME | 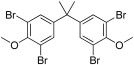 | Tetrabromobisphenol A bismethyl ether | 571.92 | 10.35 | 1.00E + 07 | na | 2.25E-06 |
| 37853-59-1 | BTBPE | 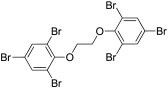 | 1,2-Bis(2,4,6-tribromophenoxy)ethane | 687.64 | 8.31 | 7.92E + 05 | na | na |
| 84852-53-9 | DBDPE | 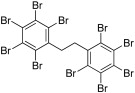 | Decabromodiphenyl ethane | 971.22 | 11.1 | 1.00E + 07 | na | na |
|  | PBDE | 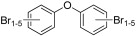 | Polybrominated diphenyl ethers |  |  |  |  |  |
| 3322-93-8 | DBE-DBCH  (α, β) | 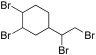 | 4-(1,2-Dibromoethyl)-1,2-dibromocyclohexane | 427.8 | 4.82 | 10,000 | na | 2.97E-03 |
| 51936-55-1 | DBHCTD | 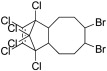 | 5,6-Dibromo-1,10,11,12,13,13-hexachloro-11-tricyclo[8.2.1.02,9]tridecene | 540.76 | 7.62 | 3.32E + 05 | na | 8.27E-07 |
| 3194-55-6 | HBCDD  ( α, β, γ) | 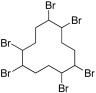 | 1,2,5,6,9,10-Hexabromocyclododecane | 641.7 | 7.92 | 4.86E + 05 | na | 1.04E-07 |
| 31107-44-5 | DDC-DBF  (Dec 602) | 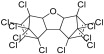 | 1,2,3,4,6,7,8,9,10,10,11,11-Dodecachloro-1,4,4a,5a,6,9,9a,9b-octahydro-1,4:6,9-dimethanodibenzofuran; Dechlorane 602 | 613.62 | 8.3 | 7.78E + 05 | na | 1.48E-09 |
| 13560-89-9 | DDC-CO  (syn and anti) | 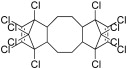 | Dodecachlorodimethanodibenzocyclooctane; Dechlorane Plus | 653.72 | 10.12 | 7.67E + 06 | na | 1.37E-11 |
| 13560-92-4 | DDC-Ant  (Dec 603) | 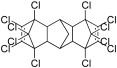 | 1,2,3,4,5,6,7,8,12,12,13,13-Dodecachloro-1,4,4a,5,8,8a,9,9a,10,10a-decahydro-1,4:5,8:9,10-Trimethanoanthracene; Dechlorane 603 | 637.68 | 9.14 | 2.24E + 06 | na | 9.16E-10 |

Table S2. GPC injection program.

| **Mobile phase** | 100 % DCM |
| --- | --- |
| **Flow rate** | 1 mL/min |
| **Injection loop** | 2000 µL |
| **Injection volume** | 950 µL |
| **Injection solvent** | 100 % DCM |
| **Columns** | 2 Agilent PLGel columns in series: PLgel particle diameter 5 µm, pore size 50 Å, 300 x 7.5 mm |
| **Column temp** | 20 °C |
| **Detection** | PDA 254 nm |

Table S3. Partisil injection program.

| **Mobile phase** | Gradient containing hexane, DCM, and methanol |
| --- | --- |
| **Flow rate** | 0.5 mL/min |
| **Injection loop** | 2000 µL |
| **Injection volume** | 400 µL |
| **Injection solvent** | 100 % hexane |
| **Column** | 1 Interchim Partisil column: PAC5, particle diameter 5 µm, 250 x 4.0 mm with pre-column of the same phase |
| **Column temp** | 20 °C |
| **Detection** | PDA 254 & 235 nm |

Table S4. Mobile phase composition of the HPLC fractionation on the Partisil column.

| **Time (min)** | **%Hexane** | **%DCM** | **%Methanol** |
| --- | --- | --- | --- |
| 0 | 100 | 0 | 0 |
| 10 | 100 | 0 | 0 |
| 15 | 80 | 20 | 0 |
| 20 | 0 | 100 | 0 |
| 30 | 0 | 100 | 0 |
| 35 | 0 | 95 | 5 |
| 60 | 0 | 95 | 5 |
| 65 | 0 | 100 | 0 |
| 70 | 100 | 0 | 0 |

Table S5. GC mass spectrometer optimised parameters, range of calibration concentrations, correlation coefficient (r) and instrumental limit of detection (ILOD) defined as 3 times the signal-to-noise (S/N) ratio calculated from MassHunter software using the root-mean-square (RMS) algorithm and a noise standard deviation multiplier of 5.

| **Compound** | **Target Transition** | **Collision Energy eV** | **Confirmation Transition** | **Collision Energy eV** | **Confirmation Transition 2** | **Collision Energy eV** | **Calibrate concentration**  **(ng ml^-1^)** | **r** | **ILOD (pg)** |
| --- | --- | --- | --- | --- | --- | --- | --- | --- | --- |
| TCMX | 244.0 -> 209.0 | 15 | 171.0 -> 136.0 | 15 |  |  |  |  | 0.1 |
| BDE 17 | 406.0 -> 246.0 | 20 | 406.0 -> 167.0 | 25 | 408.0 -> 248.0 | 22 | 6.25-400 | 0.999 | 0.5 |
| BDE 28 | 406.0 -> 246.0 | 20 | 406.0 -> 167.0 | 25 | 408.0 -> 248.0 | 22 | 6.25-400 | 0.998 | 0.7 |
| BDE 71 | 484.0 -> 324.0 | 32 | 325.0 -> 217.0 | 30 | 327.0 -> 219.0 | 30 | 6.25-400 | 0.998 | 1.2 |
| BDE 47 | 484.0 -> 324.0 | 32 | 325.0 -> 217.0 | 30 | 327.0 -> 219.0 | 30 | 6.25-400 | 0.998 | 0.8 |
| BDE 66 | 484.0 -> 324.0 | 32 | 325.0 -> 217.0 | 30 | 327.0 -> 219.0 | 30 | 6.25-400 | 0.996 | 1.5 |
| BDE 100 | 404.0 -> 297.0 | 20 | 564.0 -> 404.0 | 35 | 406.0 -> 299.0 | 35 | 6.25-400 | 0.994 | 0.7 |
| BDE 99 | 404.0 -> 297.0 | 20 | 564.0 -> 404.0 | 35 | 406.0 -> 299.0 | 35 | 6.25-400 | 0.997 | 0.8 |
| BDE 85 | 404.0 -> 297.0 | 20 | 564.0 -> 404.0 | 35 | 406.0 -> 299.0 | 35 | 6.25-400 | 0.995 | 2 |
| BDE 154 | 484.0 -> 324.0 | 20 | 644.0 -> 484.0 | 40 |  |  | 6.25-400 | 0.996 | 2.2 |
| BDE 153 | 484.0 -> 324.0 | 20 | 644.0 -> 484.0 | 40 |  |  | 6.25-400 | 0.997 | 1.7 |
| BDE 138 | 644.0 -> 484.0 | 20 | 484.0 -> 324.0 | 40 |  |  | 6.25-400 | 0.997 | 3.6 |
| BDE 183 | 562.0 -> 455.0 | 17 | 722.0 -> 562.0 | 45 |  |  | 6.25-400 | 0.993 | 8.3 |
| BDE 190 | 564.0 -> 457.0 | 45 |  |  |  |  | 6.25-400 | 0.996 | 13 |
| BDE 209 | 799.4 -> 799.4 | 15 | 799.4 -> 639.7 | 45 | 797.5 -> 637.7 | 45 | 62.5-2000 | 0.991 | 19 |
| TBP-AE | 369.8 -> 210.0 | 10 | 369.8 -> 131.0 | 25 |  |  | 10-400 | 0.999 | 0.9 |
| DBE-DBCH-α | 267.1 -> 105.1 | 15 | 187.1 -> 105.1 | 15 |  |  | 10-400 | 0.999 | 1.1 |
| DBE-DBCH-β | 267.1 -> 105.1 | 15 | 187.1 -> 105.1 | 15 |  |  | 10-400 | 0.999 | 1.5 |
| PBB | 474.2 -> 313.9 | 10 | 474.2 -> 392.8 | 45 | 474.2 -> 474.2 | 30 | 6.25-400 | 0.993 | 1.6 |
| PBT | 485.6 -> 246.9 | 20 | 485.6 -> 406.8 | 55 |  |  | 10-400 | 0.998 | 0.2 |
| PBEB | 499.7 -> 484.8 | 20 | 499.7 -> 324.8 | 50 |  |  | 10-400 | 0.999 | 0.5 |
| HBB | 552.2 -> 552.2 | 10 | 552.2 -> 391.5 | 45 | 552.2 -> 472.5 | 25 | 6.25-400 | 0.996 | 7.9 |
| PBB-Acr | 476.9 -> 448.9 | 5 | 476.7 -> 369.8 | 15 |  |  | 6.25-400 |  | 20 |
| Dec 602 | 272.0 -> 236.9 | 15 | 272.0 -> 140.9 | 35 | 272.0 -> 234.9 | 15 | 6.25-400 | 0.999 | 0.1 |
| DBHCTD | 268.1 -> 107.1 | 5 | 344.8 -> 236.8 | 10 |  |  | 10-400 | 0.996 | 0.1 |
| EHTBB | 420.9 -> 392.8 | 20 | 420.9 -> 311.9 | 40 |  |  | 10-400 | 0.990 | 3.5 |
| TBBPA-BME | 556.9 -> 291.0 | 25 | 556.9 -> 273.1 | 55 | 556.9 -> 382.1 | 30 | 10-400 | 0.993 | 1.2 |
| Dec 603 | 262.8 -> 262.8 | 15 | 262.8 -> 192.9 | 35 | 262.8 -> 190.9 | 35 | 6.25-400 | 0.997 | 0.3 |
| BTBPE | 356.9 -> 251.9 | 15 | 356.9 -> 277.9 | 25 |  |  | 10-400 | 0.997 | 11 |
| BEH-TEBP | 464.9 -> 220.9 | 40 | 464.9 -> 380.7 | 55 |  |  | 10-400 | 0.999 | 12.5 |
| DDC-CO syn | 272.0 -> 236.9 | 15 | 272.0 -> 117.0 | 45 | 272.0 -> 234.9 | 15 | 6.25-400 | 0.999 | 0.1 |
| DDC-CO anti | 272.0 -> 236.9 | 15 | 272.0 -> 117.0 | 45 | 272.0 -> 234.9 | 15 | 6.25-400 | 0.998 | 0.1 |
| DBDPE | 487.1 -> 326.8 | 45 | 487.1 -> 407.6 | 20 | 487.1 -> 405.7 | 20 | 62.5-2000 | 0.998 | 46 |
|  |  |  |  |  |  |  |  |  |  |
| ^13^C_12_ BDE 77 | 498.0 -> 338.0 | 30 |  |  |  |  |  |  | 0.1 |
| ^13^C_12_ BDE 118 | 578.0 -> 416.0 | 35 |  |  |  |  |  |  | 0.4 |
| ^13^C_12_ BDE 183 | 574.0 -> 574.0 | 5 |  |  |  |  |  |  | 0.5 |

Table S6. LC mass spectrometer optimised parameters (MRM mode, dwell time of 24 ms), range of calibration concentrations, correlation coefficient (r) and instrumental limit of detection (ILOD) defined as 3 times the signal-to-noise (S/N) ratio.

| **Compound** | **Cone voltage (V)** | **Target transition** | **Collision energy (eV)** | **Confirmation transition 1** | **Collision energy (eV)** | **Confirmation transition 2** | **Collision energy (eV)** | **Calibrate concentration**  **(ng ml^-1^)** | **r** | **ILOD**  **(pg)** |
| --- | --- | --- | --- | --- | --- | --- | --- | --- | --- | --- |
| Bisphenol A | 45 | 227>212 | 18 | 227>133 | 25 | / | / | 3.9-2000 | 0.999 | 1 |
| TBP | 50 | 328.9>78.8 | 30 | 328.9>80.8 | 30 | / | 30 | 3.9-2000 | 0.998 | 0.7 |
| TBBPA | 60 | 540.7>417.8 | 40 | 542.6>419.7 | 40 | / | / | 3.9-2000 | 0.996 | 0.2 |
| α-HBCDD | 20 | 640.8>80.8 | 20 | 642.8>80.8 | 20 | 640.8>78.8 | 20 | 3.9-2000 | 0.998 | 0.4 |
| β-HBCDD | 20 | 640.8>80.8 | 20 | 642.8>80.8 | 20 | 640.8>78.8 | 20 | 3.9-2000 | 0.998 | 0.3 |
| γ-HBCDD | 20 | 640.8>80.8 | 20 | 642.8>80.8 | 20 | 640.8>78.8 | 20 | 3.9-2000 | 0.998 | 0.3 |
| ^13^C_12_TBBPA | 60 | 554.8>459.9 | 40 |  |  | / | / |  |  |  |
| d_18_ α-HBCDD | 20 | 657.8>80.8 | 20 | 659.9>80.8 | 20 | / | / |  |  |  |
| d_18_ β-HBCDD | 20 | 657.8>80.8 | 20 | 659.9>80.8 | 20 | / | / |  |  |  |
| d_18_ γ-HBCDD | 20 | 657.8>80.8 | 20 | 659.9>80.8 | 20 | / | / |  |  |  |

Table S7. Summary of percent recovery, repeatability, reproducibility and MDL of the two treatment procedures for 4 g of sample. MDL calculated as the average of blanks with 3 times standard deviation.

|  |  | | | **H_2_SO_4_-SILICA** | | |  | | **GPC-PARTISIL** | | |  |
| --- | --- | --- | --- | --- | --- | --- | --- | --- | --- | --- | --- | --- |
|  | Average  Recoveries (%) | | Repeatability  RSD (%)  n=3 | | Reproducibility RSD(%)  n=7 | MDL  (ng g-1 dw) | Average  Recoveries (%) | Repeatability  RSD(%)  n=3 | | Reproducibility  RSD(%)  n=7 | MDL  (ng g^-1^ dw) | |
| BDE 17 | | 84 | 4 | | 22 | 0.01 | 89 | 9 | | 20 | 0.02 | |
| BDE 28 | | 84 | 8 | | 23 | 0.01 | 95 | 11 | | 23 | 0.03 | |
| BDE 71 | | 95 | 6 | | 15 | 0.01 | 106 | 11 | | 20 | 0.04 | |
| BDE 47 | | 100 | 6 | | 16 | 0.06 | 109 | 9 | | 28 | 0.06 | |
| BDE 66 | | 102 | 6 | | 9 | 0.01 | 120 | 10 | | 19 | 0.05 | |
| BDE 100 | | 96 | 5 | | 45 | 0.06 | 117 | 9 | | 44 | 0.06 | |
| BDE 99 | | 102 | 3 | | 30 | 0.07 | 117 | 9 | | 27 | 0.07 | |
| BDE 85 | | 112 | 6 | | 20 | 0.05 | 126 | 10 | | 20 | 0.05 | |
| BDE 154 | | 100 | 4 | | 19 | 0.04 | 113 | 9 | | 24 | 0.05 | |
| BDE 153 | | 117 | 5 | | 25 | 0.03 | 126 | 11 | | 15 | 0.05 | |
| BDE 138 | | 124 | 3 | | 43 | 0.04 | 147 | 11 | | 41 | 0.06 | |
| BDE 183 | | 121 | 11 | | 19 | 0.05 | 99 | 8 | | 17 | 0.05 | |
| BDE 190 | | 135 | 15 | | 23 | 0.12 | 223 | 9 | | 34 | 0.11 | |
| BDE 209 | | 173 | 21 | | 25 | 8.6 | 118 | 126 | | 58 | 11.8 | |
| TBP-AE | | 0 | - | | - | - | 123 | 12 | | 12 | 0.05 | |
| DBE-DBCH-α | | 144 | 8 | | 27 | 0.17 | 134 | 18 | | 24 | 0.36 | |
| DBE-DBCH-β | | 138 | 5 | | 24 | 0.73 | 126 | 14 | | 21 | 1.01 | |
| PBB | | 108 | 6 | | 23 | 0.48 | 125 | 14 | | 29 | 1.31 | |
| PBT | | 106 | 7 | | 22 | 0.02 | 118 | 9 | | 28 | 0.04 | |
| PBEB | | 106 | 9 | | 22 | 0.02 | 116 | 10 | | 27 | 0.09 | |
| HBB | | 97 | 15 | | 17 | 0.48 | 126 | 13 | | 28 | 0.24 | |
| PBB-Acr | | 0 | - | | - | - | 213 | 5 | | 36 | 0.82 | |
| Dec 602 | | 70 | 4 | | 15 | 0.03 | 70 | 14 | | 13 | 0.24 | |
| DBHCTD | | 86 | 6 | | 30 | 0.04 | 119 | 5 | | 26 | 0.23 | |
| EHTBB | | 122 | 11 | | 30 | 0.11 | 179 | 20 | | 24 | 0.26 | |
| TBBPA-BME | | 0 | - | | - | - | 100 | 18 | | 11 | 0.04 | |
| Dec 603 | | 108 | 6 | | 21 | 0.09 | 93 | 15 | | 29 | 0.11 | |
| BTBPE | | 261 | 8 | | 18 | 0.12 | 329 | 18 | | 45 | 0.31 | |
| BEH-TEBP | | 0 | - | | - | - | 235 | 27 | | 33 | 0.76 | |
| DDC-CO syn | | 125 | 8 | | 20 | 0.06 | 108 | 18 | | 31 | 0.08 | |
| DDC-CO anti | | 126 | 8 | | 18 | 0.07 | 95 | 19 | | 44 | 0.26 | |
| DBDPE | | 99 | 3 | | 22 | 7.6 | 91 | 57 | | 47 | 10.8 | |
| Bisphenol A | | 0 | - | | - | - | 0 | - | | - | - | |
| TBP | | 4007 | 37 | | 207 | 0.02 | 896 | 41 | | 106 | 0.03 | |
| TBBPA | | 239 | 26 | | 38 | 0.01 | 113 | 32 | | 21 | 0.01 | |
| α-HBCDD | | 81 | 16 | | 30 | 0.004 | 25 | 34 | | 54 | 0.011 | |
| β-HBCDD | | 122 | 36 | | 25 | 0.003 | 106 | 7 | | 6 | 0.015 | |
| γ-HBCDD | | 58 | 10 | | 34 | 0.003 | 57 | 24 | | 31 | 0.002 | |

Table S8. Concentrations (ng g^-1^ dw) of the target HFRs in IAEA reference materials (under preparation or certified for other elements, (e.g, trace elements, radionuclides, and other POPs)) and NIST 2974a.

|  | MUSSEL | FISH | FISH | FISH | MUSSEL | FISH | CLAM | MUSSEL | MUSSEL |
| --- | --- | --- | --- | --- | --- | --- | --- | --- | --- |
|  | North S | Aquac. | North S | Med. S | Med. S | North S. | Caledonia | measured | Certified^1^ |
|  | 2002 | 2000 | 2010 | 2004 | 2003 | 2010 | 2009 | values | values |
|  | IAEA-432 | IAEA-406 | IAEA-415 | IAEA-435 | IAEA-437 | IAEA-442 | IAEA-451 | NIST 2974a | NIST 2974a |
| Lipids, mg g^-1^ dw | 63 | 119 | 139 | 164 | 66 | 135 | 67 |  |  |
| BDE 17 | <0.02 | 0.09 | <0.02 | 0.38 | 0.04 | <0.02 | 0.06 | 0.59 | 0.534±0.062 |
| BDE 28 | <0.03 | 0.54 | 0.18 | 1.47 | 0.19 | 0.13 | 0.05 | 0.94 | 0.905±0.051^1^ |
| BDE 71 | 0.07 | 1.06 | 0.52 | 0.79 | 0.31 | 0.55 | <0.26 | 1.88 |  |
| BDE 47 | 0.28 | 7.33 | 1.53 | 23.3 | 1.85 | 1.93 | 1.00 | 17.9 | 14.3±2.8 |
| BDE 66 | 0.10 | 0.79 | <0.05 | 1.72 | <0.05 | <0.05 | <0.05 | 0.42 | 0.34±0.14 |
| BDE 100 | 0.15 | 0.80 | <0.06 | 4.97 | 0.37 | 0.26 | <0.06 | 1.99 | 2.83±0.56 |
| BDE 99 | 0.17 | 1.87 | 0.40 | 0.60 | 0.94 | 0.37 | 0.89 | 5.24 | 4.78±0.24^1^ |
| BDE 85 | 0.10 | 0.11 | <0.05 | <0.05 | <0.05 | <0.05 | <0.05 | 0.31 | 0.358±0.054 |
| BDE 154 | <0.05 | 0.32 | <0.05 | 3.81 | 0.18 | <0.05 | <0.05 | 0.27 | 0.297±0.014 |
| BDE 153 | <0.05 | 0.41 | 0.15 | 0.54 | 0.07 | <0.05 | 0.13 | 0.25 | 0.201±0.014^1^ |
| BDE 138 | <0.06 | <0.06 | <0.06 | <0.06 | <0.06 | <0.06 | <0.06 | <0.06 |  |
| BDE 183 | 0.08 | <0.05 | <0.05 | <0.05 | <0.05 | <0.05 | <0.05 | 0.21 |  |
| BDE 190 | <0.11 | 0.74 | 0.20 | 0.32 | 0.17 | 0.11 | 0.28 | 0.23 |  |
| BDE 209 | <11.8 | <11.8 | <11.8 | <11.8 | <11.8 | <11.8 | <11.8 | <11.8 | 1.99±0.11^1^ |
| ∑TetraBDE (71, 47& 66) | 0.45 | 9.17 | 2.05 | 19.6 | 2.16 | 2.49 | 1.00 |  |  |
| ∑PentaBDE (100, 99&85) | 0.42 | 2.68 | 0.40 | 5.07 | 1.31 | 0.63 | 0.89 |  |  |
| ∑BDE -28,47,99, 100,153, & 154 | 0.72 | 11.27 | 2.26 | 26.7 | 3.60 | 2.70 | 2.07 |  |  |
| TBP-AE | <0.05 | <0.05 | <0.05 | <0.05 | <0.05 | <0.05 | <0.05 |  |  |
| DBE-DBCH-α | 1.94 | 1.19 | 13.67 | 8.82 | 7.15 | 52.05 | 2.49 |  |  |
| DBE-DBCH--β | 1.44 | <1.01 | 9.37 | 6.52 | 5.18 | 36.19 | 1.69 |  |  |
| PBB | <1.31 | <1.31 | <1.31 | <1.31 | <1.31 | <1.31 | <1.31 |  |  |
| PBT | <0.04 | <0.04 | <0.04 | <0.04 | <0.04 | <0.04 | <0.04 |  |  |
| PBEB | <0.09 | <0.09 | <0.09 | <0.09 | <0.09 | <0.09 | <0.09 |  |  |
| HBB | <0.24 | <0.24 | <0.24 | <0.24 | <0.24 | <0.24 | <0.24 |  |  |
| PBB-Acr | <0.82 | <0.82 | <0.82 | <0.82 | <0.82 | <0.82 | <0.82 |  |  |
| Dec 602 | <0.24 | <0.24 | <0.24 | <0.24 | <0.24 | <0.24 | <0.24 |  |  |
| DBHCTD | <0.23 | <0.23 | <0.23 | <0.23 | <0.23 | <0.23 | <0.23 |  |  |
| EHTBB | <0.26 | <0.26 | <0.26 | <0.26 | <0.26 | <0.26 | <0.26 |  |  |
| TBBPA-BME | <0.04 | <0.04 | <0.04 | <0.04 | <0.04 | <0.04 | <0.04 |  |  |
| Dec 603 | 0.16 | 0.29 | <0.11 | <0.11 | <0.11 | 0.15 | 0.37 |  |  |
| BTBPE | <0.31 | <0.31 | <0.31 | <0.31 | <0.31 | <0.31 | <0.31 |  |  |
| BEH-TEBP | <0.76 | <0.76 | <0.76 | <0.76 | <0.76 | <0.76 | 1.41 |  |  |
| DDC-CO syn | <0.08 | <0.08 | <0.08 | 0.10 | <0.08 | <0.08 | <0.08 |  |  |
| DDC-CO-anti | <0.26 | <0.26 | <0.26 | <0.26 | <0.26 | <0.26 | <0.26 |  |  |
| DBDPE | <10.8 | <10.8 | <10.8 | <10.8 | <10.8 | <10.8 | <10.8 |  |  |
| Bisphenol A | <0.04 | 0.20 | 0.06 | 0.32 | 0.18 | <0.04 | <0.04 |  |  |
| TBP | 8.8 | 2.7 | 0.68 | 0.03 | 6.9 | 0.67 | 27.3 |  |  |
| TBBPA | <0.01 | <0.01 | <0.01 | <0.01 | <0.01 | <0.01 | 0.04 |  |  |
| α-HBCDD | 0.02 | <0.01 | 0.04 | 0.49 | 0.49 | <0.01 | <0.01 | 0.55 | 0.555±0.074 |
| β-HBCDD | <0.02 | <0.02 | <0.02 | <0.02 | <0.02 | <0.02 | <0.02 | <0.02 | 0.063±0.020 |
| γ-HBCDD | <0.002 | <0.002 | <0.002 | <0.002 | <0.002 | <0.002 | <0.002 |  |  |


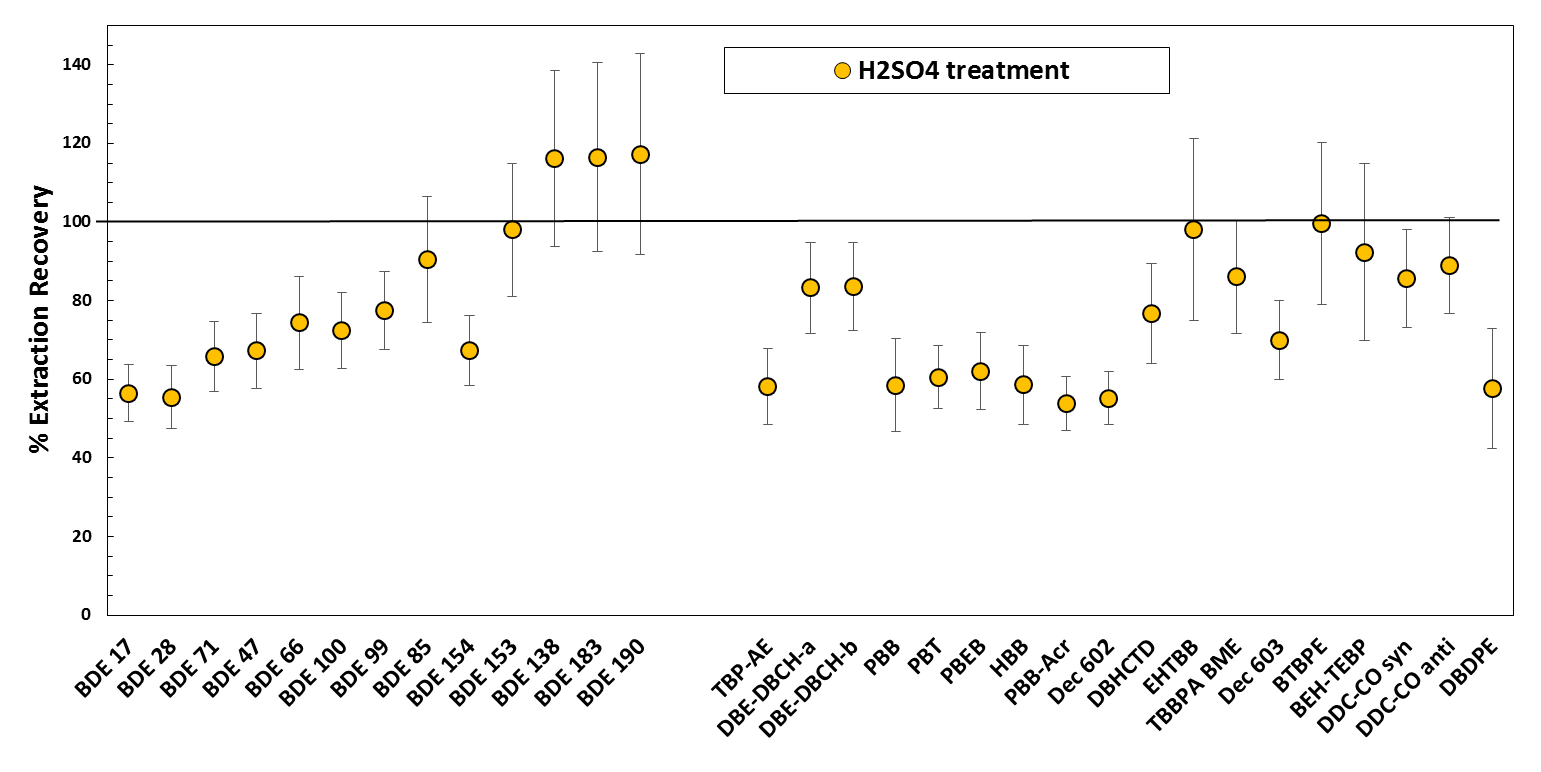


Fig. S1. Recoveries of the HFRs standard solutions after sulphuric acid treatment. The values are the mean of 3 replicates and the error bars represent the ± standard deviation.


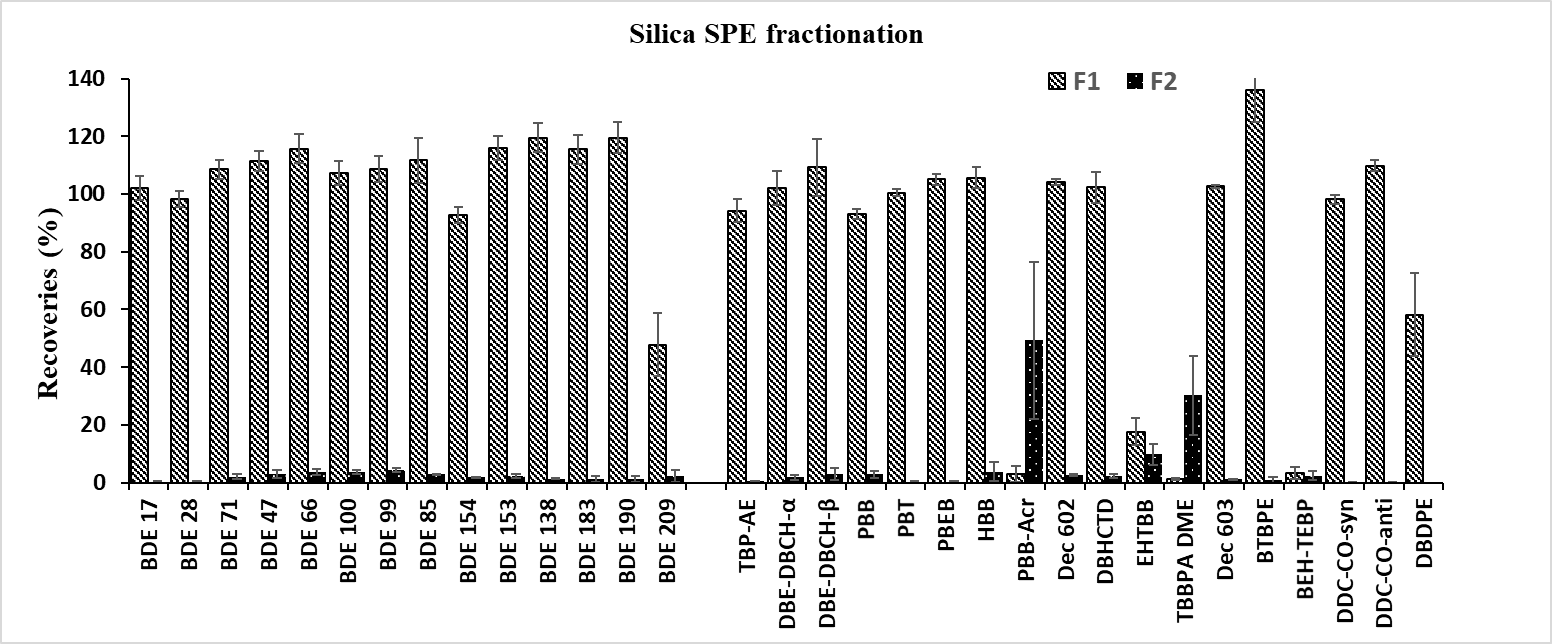


Fig. S2. Recoveries of the HFRs standard solutions after silica SPE fractionation: first fraction: 10 mL hexane:DCM (70:30); second fraction : 10 mL DCM:Methanol (50:50). The values are the mean of 3 replicates and the error bars represent the ± standard deviation.


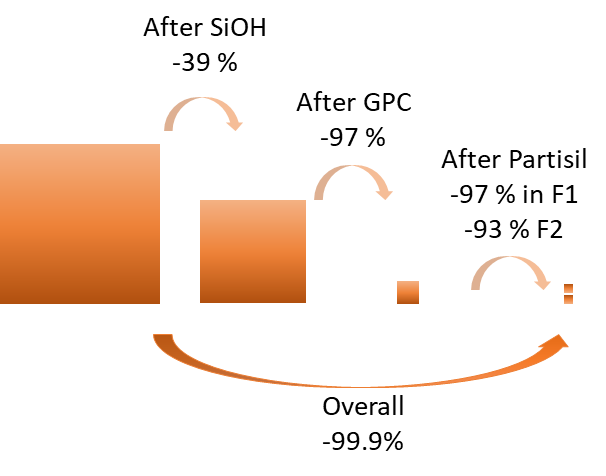


Fig. S3. Lipid removal efficiency after clean-up procedure of silica gel SPE, GPC, and Partisil HPLC column fractionation.
